# Large-Area Structural Color Filtering Capitalizing on Nanoporous Metal-Dielectric-Metal Configuration

**DOI:** 10.1186/s11671-018-2629-8

**Published:** 2018-07-20

**Authors:** Yang Li, Wen-Jing Yue, Zhen-Xiang Chen, Bing-Qiang Cao, Xiao-Qian Fu, Chun-Wei Zhang, Zhi-Ming Li

**Affiliations:** 1 School of Information Science and Engineering, University of Jinan, Jinan, 250022 China; 2 School of Materials Science and Engineering, University of Jinan, Jinan, 250022 China

**Keywords:** Structural color filtering, Fabry–Perot resonance, Plasmonic effect, Nanoporous anodic alumina, Metal–dielectric–metal configuration, Large-area, Effective medium theory

## Abstract

We present a highly efficient structural color filtering approach for large-area application, using a nanoporous anodic alumina (NAA) film overlaid with an aluminum (Al) layer on top of an optically thick Al substrate. The NAA film, consisting of a self-assembled nanopore array in a hexagonal lattice, is equivalent to a quasi-homogeneous medium according to effective medium theory. The proposed structure enables strong absorption at resonance owing to the Fabry–Perot resonance supported by the metal-dielectric-metal configuration and the plasmonic effect mediated by the top nanoporous Al layer. The reflection colors can be readily tuned by altering the NAA thickness that is determined by anodization time, thereby allowing the flexible creation of complicated color images on a single platform. By fabricating three samples with different NAA thicknesses in a large area of 2 cm × 2 cm, it is confirmed that the proposed color filtering scheme exhibits highly enhanced color purity and high reflection efficiency of up to 73%, which is superior to that generated by previously reported NAA-based approaches. The presented strategy can pave the way for the efficient fabrication of large-area color filtering devices for various potential applications, including color display devices, imaging sensors, structural color printing, and photovoltaic cells.

## Background

Color filtering technologies resorting to subwavelength structures have played a vital role in a variety of fascinating applications, such as transmissive/reflective color filters in display devices, imaging systems, chromatic polarizers, photovoltaic cells, and photorealistic structural color printing [1–10]. Structural color filtering, which involves from traditional organic dyes/pigments-based chemical filters, successfully mitigates the drawbacks of chemical filters including significant performance degradation under long-standing ultraviolet illumination and serious environmental stress. Moreover, structural color filtering exhibits salient features of flexible spectral filtering properties and stable specifications. Various schemes for achieving structural colors, particularly those that involve the use of multi-layer thin films [11–15], subwavelength-grating-enabled plasmonic or guided-mode resonance nanostructures [16–22], and metasurfaces [23], have been proposed. The fabrication of subwavelength-grating-based configurations and metasurfaces generally require complicated procedures, such as electron beam (e-beam) lithography and reactive ion etching, which are time-consuming and have high cost, and greatly limit their potential applications in large-area circumstances. Thus, multi-layer thin films, particularly the Fabry–Perot (FP) resonator with a dielectric cavity sandwiched by two metallic layers, are widely used as an alternative method. However, multiple fabrication steps are needed for the deposition of different cavity thickness for the purpose of simultaneously generating full colors on a single platform, which hinder their usage in practical applications.

To mitigate the aforementioned issues, nanoporous anodic alumina (NAA), which is one of the cost-effective porous self-assembled materials consisting of many parallel straight cylindrical nanopores perpendicular to an optically thick Al substrate, is regarded as the best candidate [24, 25]. Several strategies for generating multiple structural colors on NAA films are currently used, including covering the top surface and inner sidewall of the NAA film with carbon or dielectric material, such as TiO_2_ [26–28], or depositing metallic layers on top of an NAA film [29–32]. A FP resonance-enabled asymmetric metal-dielectric-metal (MDM) configuration can be easily constructed by simply depositing a metallic layer on top of an NAA. At resonance, a strong suppression in reflection can be observed, corresponding to a specific reflection color. The metallic layer deposited on top of a NAA, which consists of a hexagonal lattice of pores, can simultaneously enable a strong plasmonic effect, further enhancing the absorption of the structure [32, 33]. Through a simple adjustment of the geometry of NAA, such as thickness and pore diameter, the observed colors can be effectively tuned. However, the reported metal-coated NAA configurations, which use noble metals such as platinum and gold, lead to a high cost of device [29, 32]. And, the optical spectra of the reported configurations exhibit low reflection efficiencies, multiple resonances within the visible spectral band, or broad spectral bandwidths, resulting in undesired low color purity.

In this work, we demonstrate a highly efficient structural color filtering scheme for large-area applications by exploiting a simple nanoporous structure based on an NAA film overlapped with a thin aluminum (Al) layer. Vivid distinctive reflection colors can be readily tuned by simply altering the thickness of the NAA. Al is particularly applied because of its outstanding optical properties, including high reflectivity in the visible regions, low cost, and compatibility with the standard complementary metal–oxide–semiconductor fabrication process [20–22]. The individual role of each geometric parameter of the proposed structure is rigorously inspected through the finite-difference time-domain (FDTD) method. Samples with different NAA thicknesses were manufactured over a large area through a non-lithography method. The optical characteristics of the prepared samples were measured and evaluated by comparing the measured results with the simulated results.

## Methods/Experimental

### Design of the Proposed Large-Area Color Filtering Scheme

**Fig. 1:**
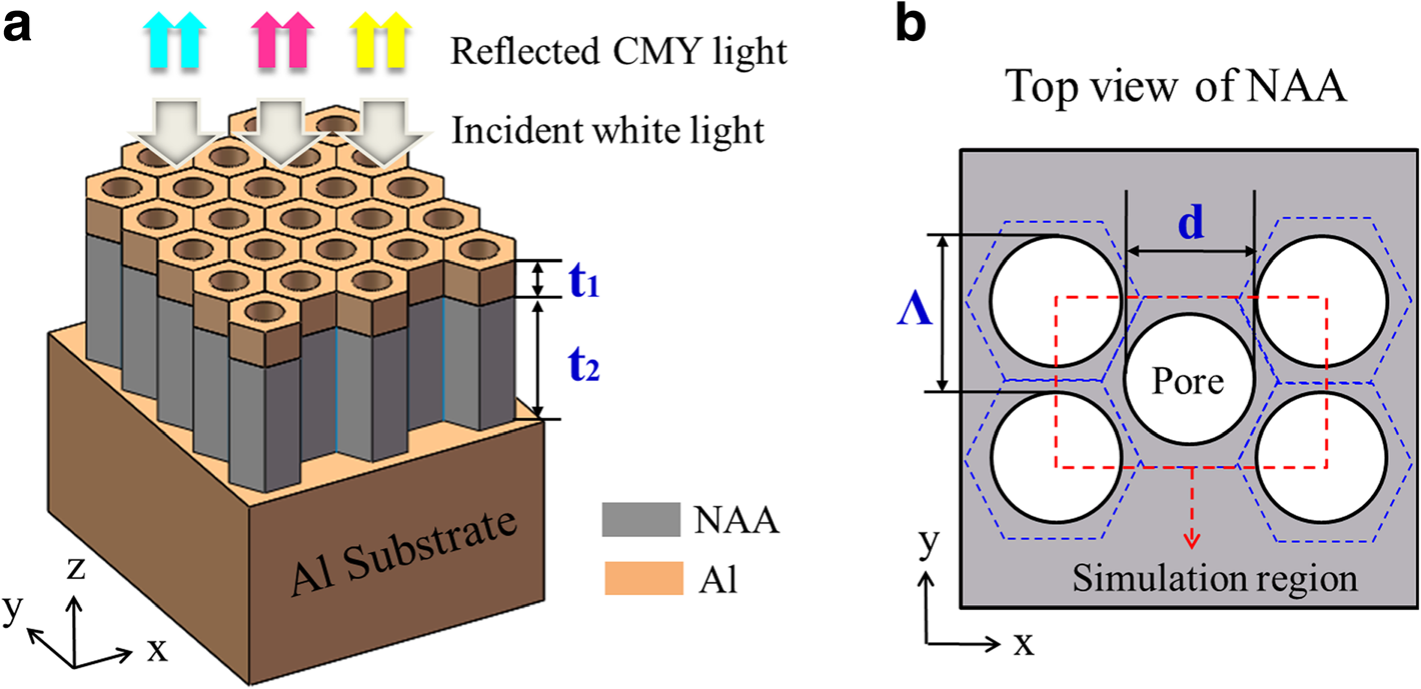
**a** Schematic geometry of the proposed structure based on the Al-coated NAA film atop an Al substrate for large-area color filtering. **b** Top view of the NAA film with a hexagonal lattice of nanopores

In this study, we aim to develop a highly efficient color filtering scheme for large-area application by capitalizing on a nanoporous MDM resonant configuration capable of supporting FP resonance in addition to the plasmonic effect. Figure 1a depicts the schematic configuration of the proposed MDM-structure-based color filtering device, where an NAA film is sandwiched between an optically thick Al substrate and a top thin Al layer. The thickness of the NAA and top Al layer are denoted as *t*_1_ and *t*_2_, respectively. For the fabrication of a nanoporous structure, the NAA film is a self-assembled porous structure that originated from an Al plate through a simple anodization process rather than through conventional approaches relying on complicated and expensive e-beam lithography. As shown in Fig. 1b, the NAA film consists of a hexagonal lattice of pores with the diameter of d. The gap between two adjacent pores is represented by *Ʌ*. When the gap between two pores is sufficiently small compared with the wavelength of interest, the nanoporous layers, embracing top Al coating and NAA film, behave as quasi-homogeneous media. Therefore, we appropriately set *Ʌ* and *d* to 100 and 65 nm, respectively. Effective medium theory has been commonly used for elucidating the properties of such nanoporous structures [34, 35].

**Fig. 2:**
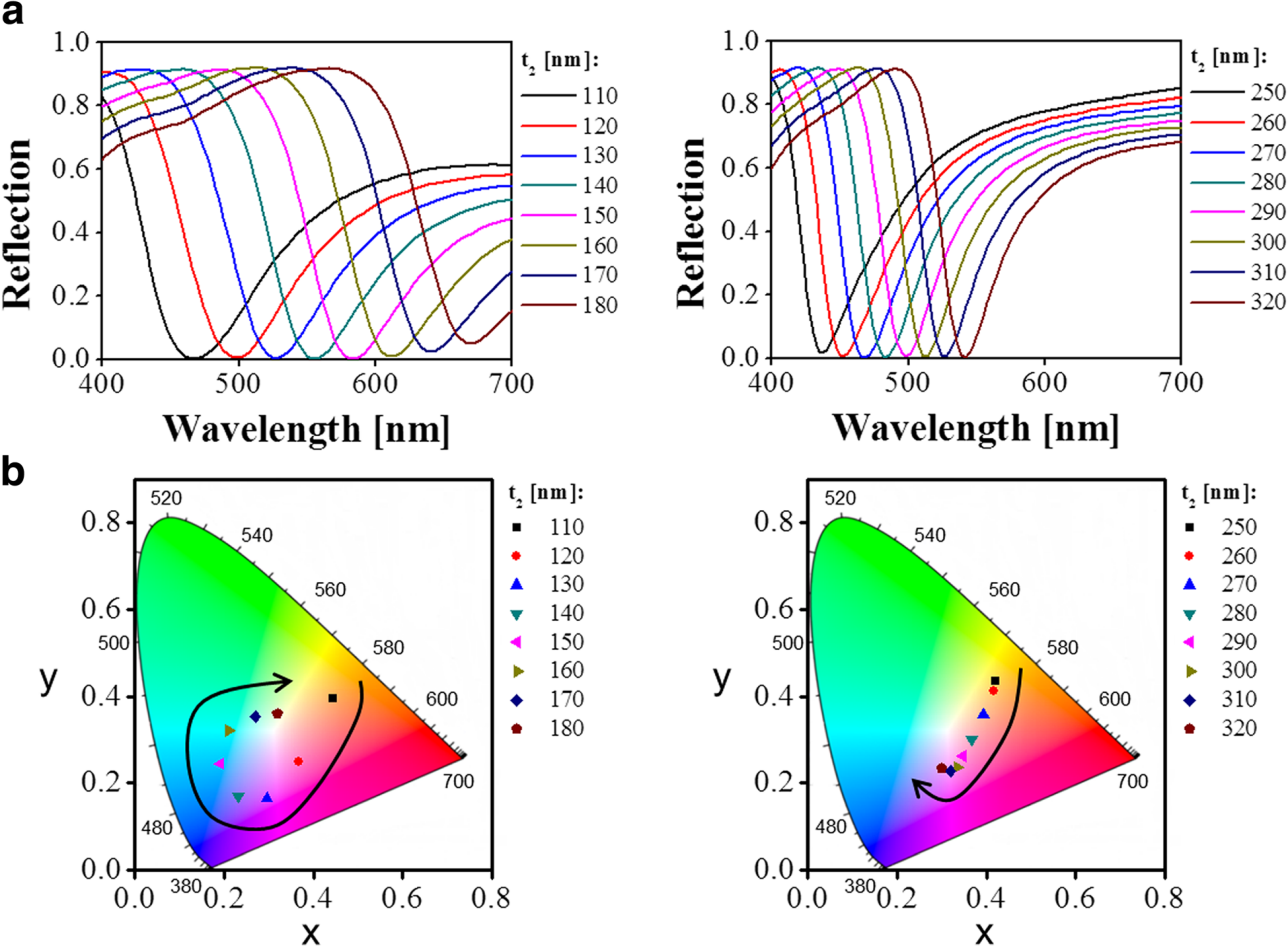
**a** Simulated reflection spectral responses of the proposed color filtering structure with NAA thickness varying from 110 to 320 nm. **b** Corresponding chromaticity coordinates in the CIE 1931 chromaticity diagram

**Fig. 3:**
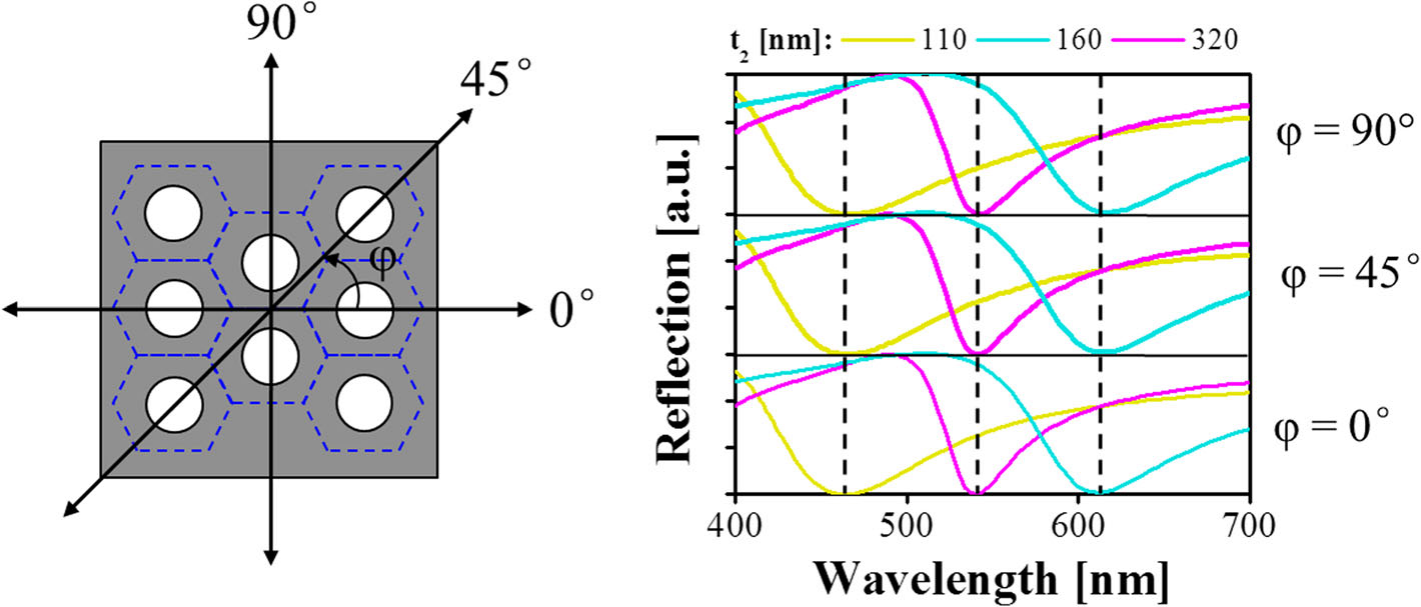
Simulated reflection spectra of the proposed structure with respect to the incident polarization

For the FP-resonance-enabled asymmetrical MDM structure, the reflection is strongly suppressed at resonance, corresponding to a reflection dip, when the deconstructive interference occurs between the directly reflected light at the top air–Al interface and the resonantly coupled light within the NAA cavity. Different from the conventional MDM structure based on continuous layers [12–14], the proposed nanoporous structure is expected to tune the reflection colors by not only changing the thickness of the dielectric cavity but also altering the pore diameter or gap [28, 29]. More importantly, owing to the top nanoporous Al layer, the proposed structure is capable of enabling a strong plasmonic effect in addition to FP resonance, which can efficiently reinforce the absorption of the proposed structure. The proposed structure is carefully designed and assessed with a tool based on the FDTD method. The dispersion characteristics of the materials used for the simulations are derived from the built-in multi-coefficient model provided by the tool. For simplicity, the simulation region, which is denoted by a dotted red box in Fig. 1b, only contains a unit structure, and the periodic boundaries are applied for the *x* and *y* axes. A default auto non-uniform mesh refinement with mesh accuracy of 3 is set for the entire simulation region. This set-up ensures a good trade-off between accuracy and simulation time. A plane wave serves as a light source. We set the thickness of the top Al coating to 15 nm through a set of simulations to obtain a near-zero reflection dip in order to produce high-purity colors. Then, the spectral tunability upon the thickness of the NAA cavity is investigated, as plotted in Fig. 2a. As the NAA thickness *t*_2_ varies from 110 to 180 nm, the resonance wavelength slightly redshifts from 465 to 670 nm, covering the entire visible spectral band. When the thickness of the NAA is further increased, the resonance dip eventually enters into the near-infrared band. Meanwhile, a higher-order resonance dip with relatively narrow bandwidth appears from the ultraviolet band to the visible band with the NAA thickness ranging from 250 to 320 nm. It should be noted that a single resonance dip in the visible band is desired for the production of vivid reflection colors with high purity. To estimate the color purity of proposed structure, the chromaticity coordinates that correspond to the reflection spectra are calculated and mapped into the standard International Commission on Illumination (CIE) 1931 chromaticity diagram, as depicted in Fig. 2b. The chromaticity coordinates are observed to evolve along the black arrow as the thickness of the NAA increases. In particular, the circular trace of the chromaticity coordinates with NAA thickness increasing from 110 to 180 nm indicates that the proposed scheme is capable of achieving vivid full colors through the simple adjustment of NAA thickness. Figure 3 depicts the polarization-dependent reflection spectra of the proposed structure with different cavity thicknesses of *t*_2_ = 110, 160, and 320 nm. It is observed that the same reflection spectra are maintained in terms of the resonance wavelength and reflection efficiency as the polarization angle of incident light varies from 0° to 90°. Therefore, the proposed structure is deemed to enable the polarization-independent property, which is attributed to the symmetrical geometry of the proposed structure.

### Fabrication of Color Filtering Devices

**Fig. 4:**
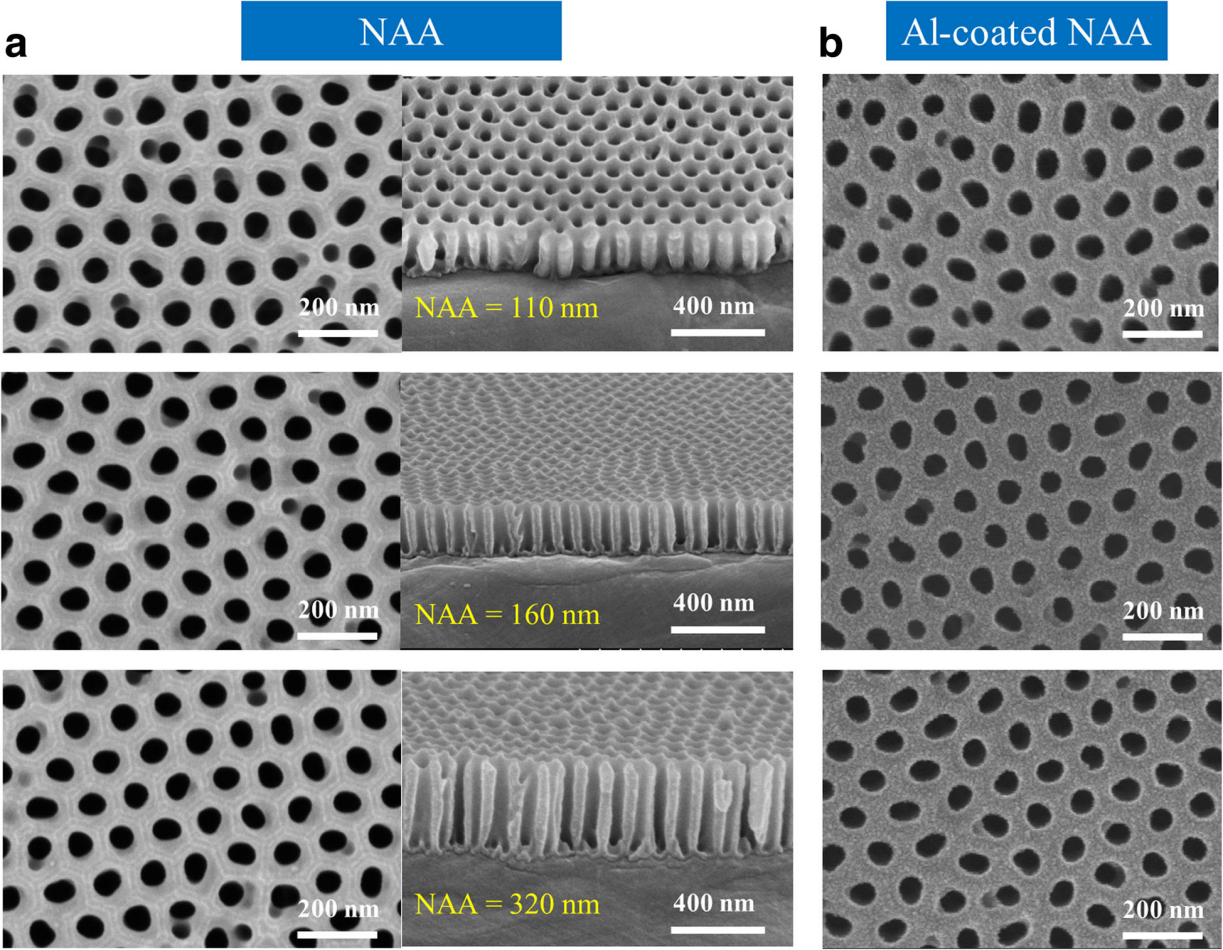
**a** Top and cross-sectional views of the SEM images of the manufactured NAA films with different thicknesses of *t*_2_ = 110, 160, and 320 nm, respectively. **b** Top view of the SEM images of the proposed structure based on Al-coated NAA film

For the purpose of evaluating the proposed color filtering scheme, we manufactured three samples with different NAA thicknesses through the following fabrication processes. Commercial high-purity (99.999%) Al foil was initially degreased in acetone and subsequently washed in isopropyl alcohol and deionized water without any other pretreatments before adonization. The prepared Al foil was cut into square pieces, which were placed in a self-made holder with an effective area of 2 cm × 2 cm during the anodization process. The electrolyte container was a transparent beaker with a total volume 4 L. In this experiment, a two-step anodization process was sequentially implemented. In the first step, anodization was performed by immersing the Al foil square pieces into 0.3 M oxalic acid under the constant anodization voltage of 40 V at room temperature for 30 min. Afterward, the anodized specimens were immersed in a mixture of 6.0 wt% H_3_PO_4_ and 1.8 wt% H_2_CrO_4_ at 60 °C for 5 h for the removal of the oxidized layers. In the second step, anodization was performed with the same experimental conditions used in the first step. As a result, the partially anodized parts of the original Al foil pieces were transformed into the NAA films with well-defined straight pores. An undesired optically thick alumina layer was formed within the pore at the top of the underlying Al foil because of the oxidation of Al during the second anodization step. In order to remove the undesired alumina layer within the pore completely, the anodized samples were dissolved in 6.0 wt% H_3_PO_4_ at 60 °C for 10 min. Finally, three samples with different NAA thicknesses of 110, 160, and 320 nm, were prepared by accurately controlling the anodization time. The top and cross-sectional views of the fabricated NAA samples are presented in Fig. 4a, exhibiting a satisfactory nanoporous structure with well-shaped pores and high periodicity. For the prepared samples, the pore diameter and gap between two neighboring pores were measured to be *d* = 65 nm and *Ʌ* = 100 nm, respectively. Then, an Al coating layer was deposited on top of the prepared NAA film via sputtering deposition under the base pressure of 6.7 × 10^−5^ Pa and 2.0 kW direct current power for 260 s. Notably, the minimum deposition rate of 0.5 Å/s was selected to ensure the thickness accuracy of the deposited Al layer. Figure 4b illustrates the top view of the scanning electron microscopy (SEM) images of the manufactured color filtering devices with thin Al coating layers on top. The thickness of the deposited Al layers was measured to be *t*_1_ = 16 nm, which is close to the designed thickness.

### Optical Characterization of the Prepared Color Filtering Devices

**Fig. 5:**
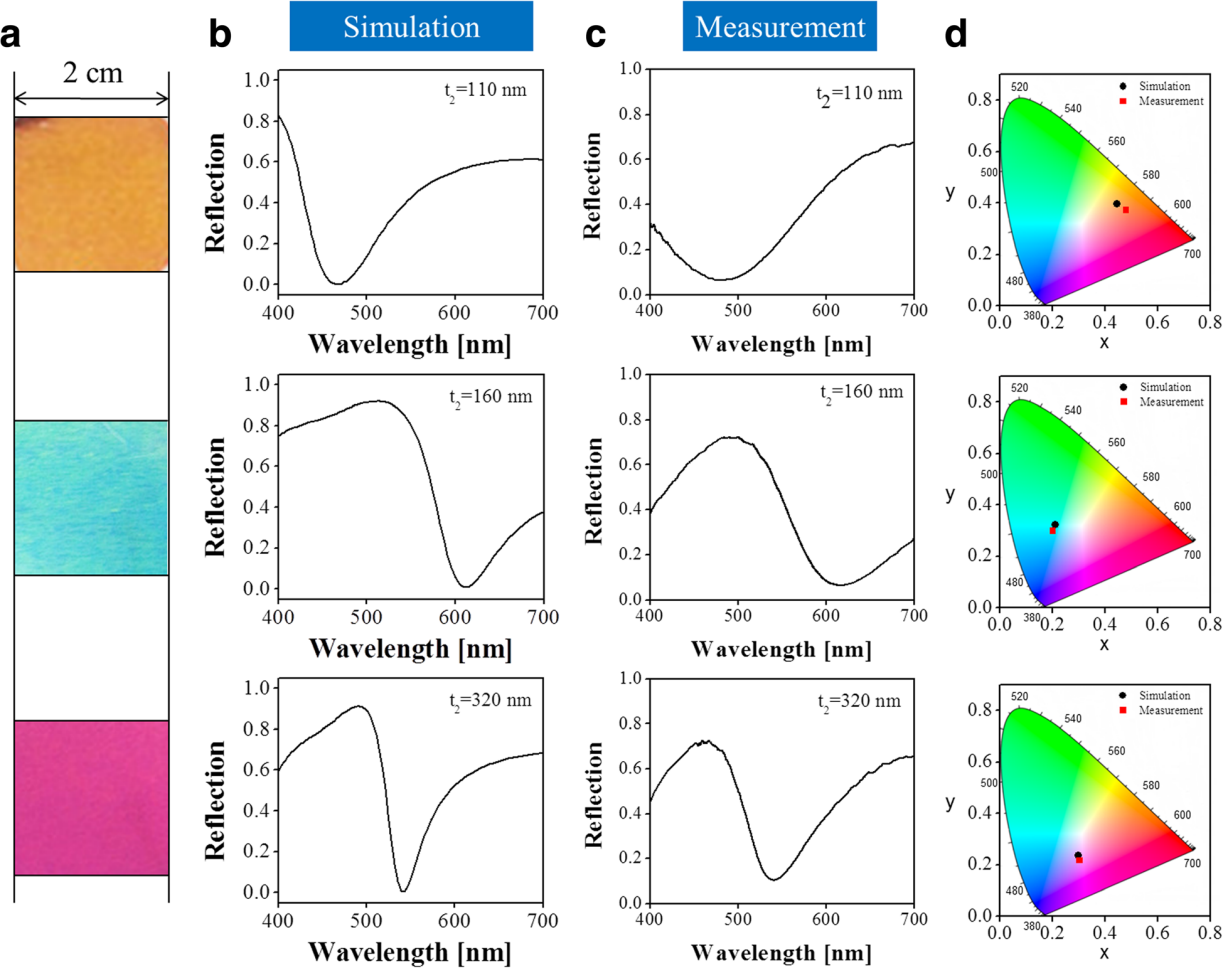
**a** Captured optical color images at normal incidence from the manufactured devices with different NAA thicknesses of *t*_2_ = 110, 160, and 320 nm. **b** Simulated and (**c**) measured reflection spectra of the fabricated devices. **d** Corresponding chromaticity coordinates in the CIE 1931 chromaticity diagram in response to the simulated and measured spectra

The optical performance of each prepared sample was thoroughly assessed with respect to reflection color and spectral response. Figure 5a shows the measured reflection colors at normal incidence from the manufactured samples with large dimensions of 2 cm × 2 cm. Vivid primary subtractive colors of yellow, cyan, and magenta were observed, verifying that the proposed color filtering approach is capable of rendering full-color generation with highly enhanced color purity. For a better understanding of the achieved high purity, a customized experimental setup, including a halogen lamp serving as the light source, a beam splitter, and a spectrometer, was implemented to measure the reflection spectra of the prepared samples. Figure 5b, c depicts the measured reflection spectra together with the simulated reflection spectra as references, where a good correlation was observed between the experiment and the simulation with regard to resonance wavelength and shapes of the reflection spectra. A small discrepancy in spectral bandwidth and reflection efficiency can be ascribed to the imperfection of the fabrication with regard to the design, including the roughness of the Al-NAA interfaces and inconsistent periodicity and size of the pores, which could be easily observed in Fig. 4. It is also found that the fabricated samples with NAA thicknesses of 110, 160, and 320 nm had near-zero resonance dips located at wavelengths of 484, 614, and 539 nm, respectively, and practically achieved high reflection efficiencies of up to 73%. The chromaticity coordinates corresponding to the simulated and measured spectra were calculated and plotted in the CIE 1931 chromaticity diagram, as depicted in Fig. 5d. The observed high-purity reflection colors shown in Fig. 5a are confirmed to benefit from the achieved high reflection efficiency and near-zero reflection dip.

## Results and Discussion

### Investigation of the Plasmonic Effect

To examine the plasmonic effect enabled by the nanoporous Al layer, we thoroughly investigate the proposed structure by replacing the NAA cavity with an equivalent homogeneous cavity exhibiting an effective refractive index. Based on effective medium theory, the effective refractive index of the NAA cavity with a pore gap of 100 nm and a pore diameter of 65 nm is derived to be *n*_eff_ = ~ 1.48, according to the equation expressed as follows:

1 $$ \left({n^2}_{\mathrm{eff}}-{n^2}_{{\mathrm{Al}}_2{\mathrm{O}}_3}\right)/\left({n^2}_{\mathrm{eff}}+2{n^2}_{{\mathrm{Al}}_2{\mathrm{O}}_3}\right)={f}_{\mathrm{air}}\left(1-{n^2}_{{\mathrm{Al}}_2{\mathrm{O}}_3}\right)/\left(1+2{n^2}_{{\mathrm{Al}}_2{\mathrm{O}}_3}\right). $$

**Fig. 6:**
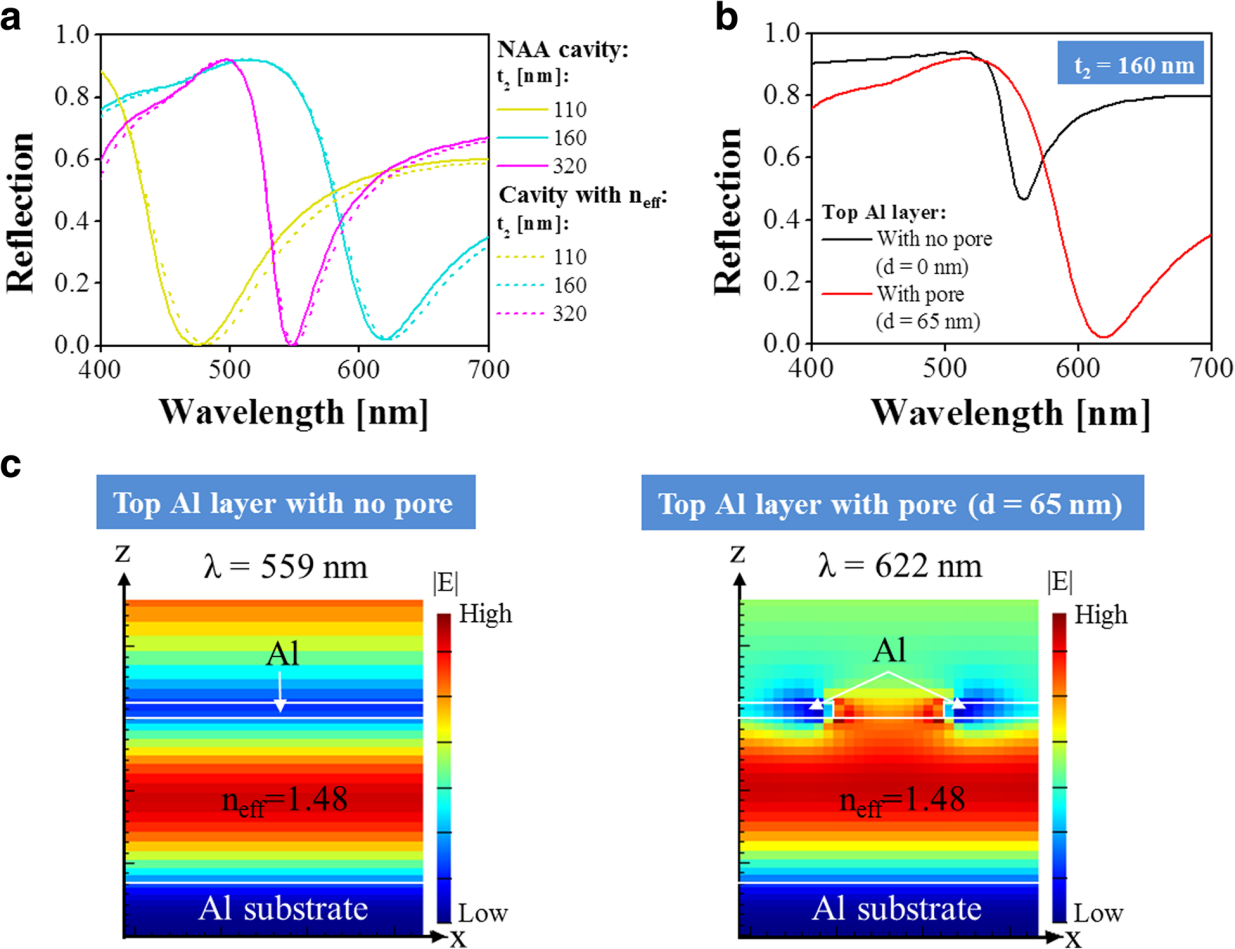
**a** Simulated reflection spectra of the proposed structure based on the NAA cavity and the equivalent structure based on a homogeneous cavity with effective refractive index (*n*_eff_) for different cavity thicknesses of *t*_2_ = 110, 160, and 320 nm. **b** Simulated reflection spectra of the structures including the top Al layer without pore and with pore (*d* = 65 nm)

Here, the refractive index of alumina (Al_2_O_3_) is *n*_Al2O3_ = 1.77 and the fill fraction of air within the NAA cavity is ${f}_{\mathrm{air}}=\pi {\left(d/\Lambda \right)}^2/2\sqrt{3}$. Figure 6a shows a comparison of the reflection spectra between the structures based on the NAA cavity and the homogeneous cavity with *n*_eff_ of 1.48 for different cavity thicknesses of *t*_2_ = 110, 160, and 320 nm. A good correlation can be observed between the two cases, indicating that the proposed structure can be safely equivalent to the structure based on a homogeneous cavity with an effective index of 1.48. For the equivalent structure based on the homogeneous cavity, the influence of pores within the top Al layer on the reflection spectrum is depicted in Fig. 6b. Compared with the case including no pore in the top Al layer, the proposed structure consisting of top Al layer with a pore diameter of *d* = 65 nm can strongly enhance the absorption at resonance. The observed clear redshift on the resonance wavelength can be ascribed to the result of the plasmonic effect and the changed phase shift in reflection at the top Al layer. To verify whether the introduced pores in the top Al layer lead to the observed absorption enhancement through the plasmonic effect, we monitor the electric field (|*E*|) profiles at resonance in the *x*–*z* plane for the two cases with and without the presence of pores on the top Al layer, as illustrated in Fig. 6c. In the structure with non-porous Al layer, despite the fact that a strong field enhancement can be observed within the cavity at the resonance wavelength of 559 nm because of the FP resonance supported by the MDM structures, part of the light is still reflected. While for the structure with porous top Al layer, it is observed that the electric field enhancement is not only within the cavity but also within the pore in the top Al layer through the plasmonic effect at the resonance wavelength of 622 nm. As a result, light is nearly completely confined within the proposed structure, corresponding to the near-zero reflection dip shown in Fig. 6b.

### Influence of the Al Oxidation

**Fig. 7:**
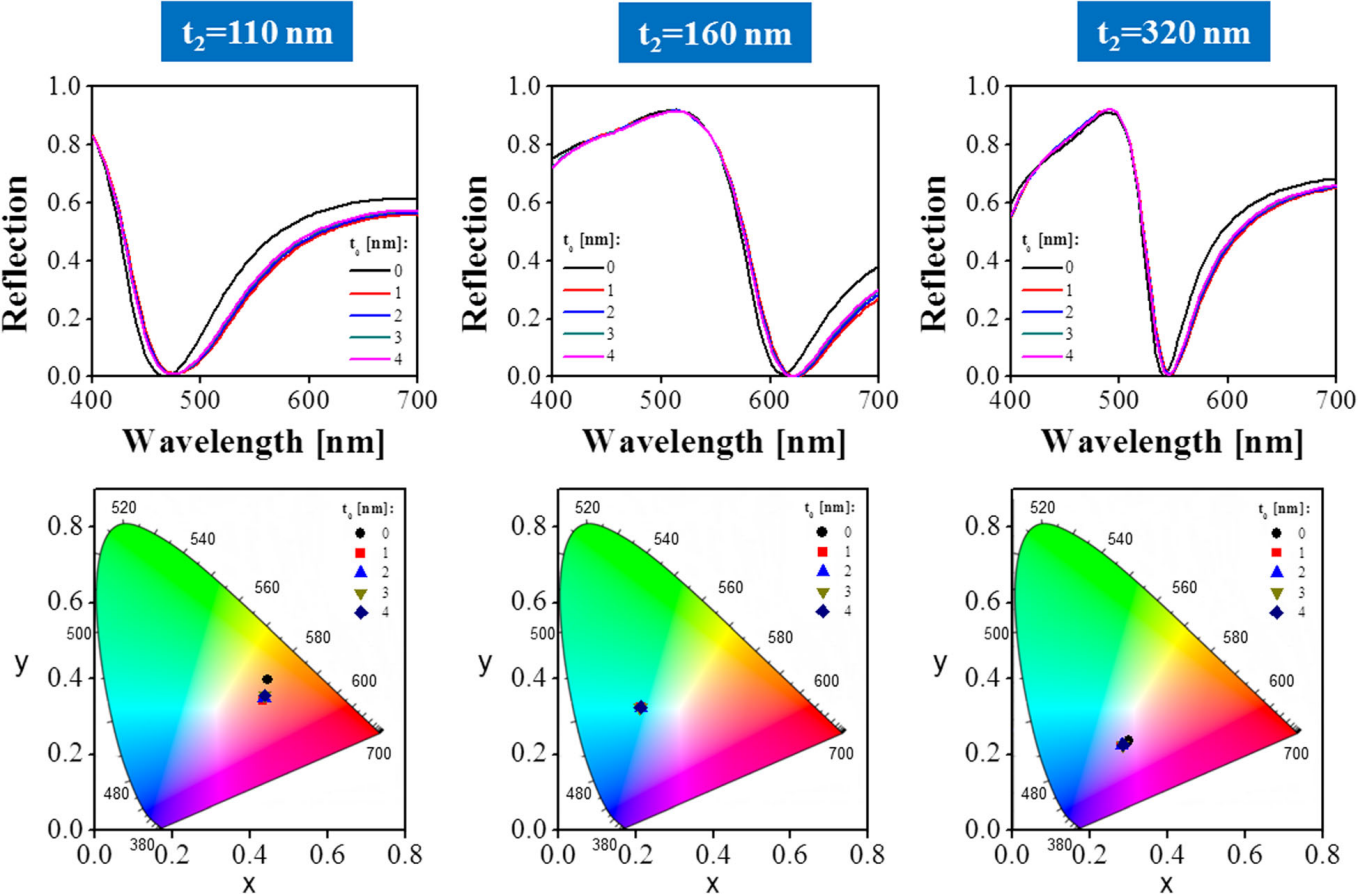
Simulated reflection spectra of the proposed structure considering the formed alumina layer on top of Al because of the air oxidation of the different NAA cavity thicknesses of 110, 160, and 320 nm

**Fig. 8:**
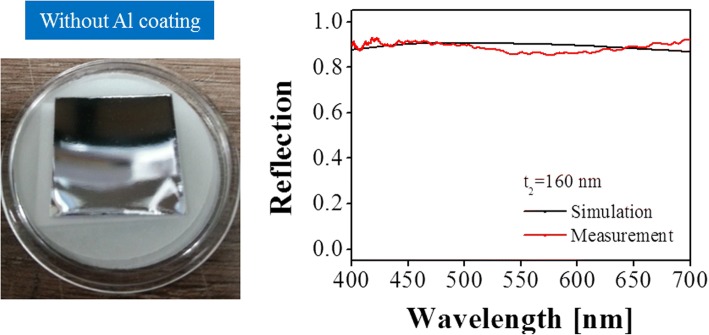
Captured optical color image of the simulated and measured reflection spectra of the reference structure with no Al coating layer on top of the NAA film

Notably, a 0.5–4 nm-thick alumina layer spontaneously formed on the surface of Al because of air oxidation of Al at room temperature [36, 37]. The alumina layer serving as a stable passive layer can protect Al from further oxidation. Taking this situation into consideration, the reflection spectra and corresponding chromaticity coordinates of the structure with different NAA thicknesses were inspected, respectively, as shown in Fig. 7. As the thickness of the alumina layer on the surface of Al layers, including the top nanoporous Al layer and the bottom Al substrate, increased from *t*_0_ = 0 to 4 nm, and the reflection spectra maintained good consistency in terms of resonance wavelength and reflection efficiency. Moreover, the chromaticity coordinates indicated a stable color output after the oxidation of Al. As a result, the air oxidation of Al hardly affected the optical performance of the proposed structure. For comparison, the structure without an Al coating layer was also evaluated. As depicted in Fig. 8, the thickness of the NAA film was 160 nm. The gray color, which is the original color of the Al foil, was observed, further confirming that the proposed color filtering scheme enabled highly enhanced color purity. As indicated by the simulated and measured reflection spectra of the structure without an Al layer on top, no obvious resonance phenomenon was observed in the visible spectral band, resulting in the observed low-purity reflection color. Notably, the appearance of the reflection spectrum of the NAA film without Al coating layer was similar regardless of the thickness of the NAA film, whereas that of the Al-coated NAA film strongly depended on thickness.

## Conclusions

In summary, we proposed and demonstrated an attractive method for achieving large-area color generation with high color purity through the use of a thin Al coating layer in conjunction with the NAA film atop an optically thick Al substrate. According to effective medium theory, the nanoporous layers belonging to the proposed structure, including the Al coating layer and NAA film, behave as the quasi-homogeneous media with certain effective refractive indices. As a result, the proposed structure operates as a MDM resonant structure that enables FP resonance, where the resonance wavelength corresponding to the reflection color is readily tuned by simply changing the NAA thickness. Meanwhile, by taking advantage of the top nanoporous Al layer, we found that the proposed structure supported the plasmonic effect, which can strongly enhance the absorption leading to the observed near-zero reflection dip. The optical performances of the proposed structure depending on its geometry were theoretically scrutinized using the FDTD-method-based tool. On the basis of optimized parameters, three samples with different NAA thicknesses were manufactured in a 2 cm × 2 cm area. Through the analysis of the experimental results, the prepared samples are verified to exhibit vivid reflection colors with high reflection efficiency of up to approximately 73%. The proposed approach can not only lead to a better understanding of the color-tuning mechanism of NAA-film-based configuration but also represent an important step toward the realization of cost-effective large-area color filtering devices in a large number of applications, such as display/imaging devices, photovoltaic cells, and biosensor technologies.

## Abbreviations

|E|: Electric field
Al: Aluminum
Al_2_O_3_: Alumina
CIE: International Commission on Illumination
e-beam: Electron beam
FDTD: Finite-difference time-domain
FP: Fabry-Perot
MDM: Metal-dielectric-metal
NAA: Nanoporous anodic alumina
SEM: Scanning electron microscopy
